# Edge Driven Trust Aware Threat Detection for IoT Enabled Intelligent Transportation Systems

**DOI:** 10.3390/s26041108

**Published:** 2026-02-09

**Authors:** Khulud Salem Alshudukhi, Mamoona Humayun, Aala Oqab Alsalem, Mohammad Farhan Khan, Khalid Haseeb

**Affiliations:** 1 Department of Computer Science, College of Computer and Information Sciences, Jouf University, Sakaka 72388, Saudi Arabia; alaa.alsalem@ju.edu.sa; 2 School of Computing, Engineering and the Built Environment, University of Roehampton, London SW15 5PH, UK; mamoona.humayun@roehampton.ac.uk (M.H.); mohammad.f.khan@roehampton.ac.uk (M.F.K.); 3 Department of Computer Science, Islamia College Peshawar, Peshawar 25120, Pakistan

**Keywords:** artificial intelligence, Internet of Things, vehicular networks, edge computing, security

## Abstract

Wireless communication and the Internet of Things (IoT) are integrated for the formulation of an emerging Intelligent Transportation System (ITS) for the interaction of vehicles and to enhance road safety. The emerging network manages the traffic flow, real-time data analytics, and resource control for the development of urban transportation systems and smart cities. Extensive research has been conducted on the development of efficient routing response time for the IoT-ITS environment; however, the rapid changes in the network topologies still lead to unmanageable congestion and communication holes. Moreover, it is also often threatened due to high urban mobility and incurs additional transmission with excessive overhead. Such concepts are not able to maintain secure interactions among vehicles and expose confidential data to malicious devices while interacting on unpredictable channels. This research proposes a trust-aware edge-assisted model to secure the vehicular network and offers a more reliable system with optimal routing performance. The global trust model is maintained based on network conditions using localized computing and attaining data privacy and coherence. Furthermore, a blockchain ledger is included along with trust to ensure tamper-proof and transparent computing across the boundaries of the IoT-ITS environment. The proposed model is compared with Graph-Based Trust-Enabled Routing (GBTR) and Bacteria for Aging Optimization Algorithm (BFOA), and the results revealed significant performance for network throughput by 50% and 62.5%, end-to-end delay by 33.3% and 37.5%, routing overhead by 34% and 38.7%, and false positive rate by 67.9% and 68.5% over the dynamic network infrastructure.

## 1. Introduction

Data-driven applications provide autonomous communication in the growth and development of intelligent systems [1,2]. IoT and edge technologies enhance the processing capabilities of the devices near the source and improve the responsiveness of the real-time applications [3]. Intelligent Transportation System (ITS) using emerging technologies improving vehicles communication, seamless connectivity, and road safety, thus enhancing traffic management in urban systems. On the other hand, artificial intelligence enables local decision-making over a dynamic ITS environment for efficient resource management. These techniques intelligently detect the routing anomalies and optimise the traffic flow with affordable network congestion [4,5]. Moreover, using AI models, ITS smartly detect the security attacks and isolates the malicious activities, resulting in providing fault-tolerant communication between the associated wireless channels [6,7]. Using predictive algorithms, traffic hazards and road accidents can be detected to manage and providing rapid emergency response. Although due to limited resources of the Internet of Things (IoT) in ITS and the dynamic nature of the wireless technologies, most of the existing approaches necessitate the development of intelligent, adaptive security models specifically designed to protect ITS communications with high network performance [8,9]. Traditional ITS-based approaches face cybersecurity threats and impose additional computing power on constrained devices. Most of them rely on cryptographic methods with excessive energy consumption while protecting data from malicious activities and unauthentic device behavior, thus leading to overhead and unmanageable congestion [10,11,12]. Moreover, vehicles interact with Roadside Unit (RSU) at the same time, thus, communication bottlenecks can cause routing holes and a single point of failure in the centralized trust-aware paradigm. Such systems lack timely decision-making and are not able to address network vulnerabilities under dynamic conditions [13,14]. In addition, the static security policies and predefined thresholds in most of the existing approaches overlook the adaptive emerging attacks and degrade the performance by privacy leakage and loss of device trust [15,16]. Edge computing supports the processing of sensitive data locally and avoids transmitting it towards a centralized location, thus resulting in preventing potential security attacks. However, the non-uniform load distribution near the network edges causes system failures with re-transmissions and an inability to cope with timely responses in critical circumstances [17,18]. Our research study is considered to implicitly address the following questions.

i. How can edge computing be explored to formulate a distributed architecture for attaining resource optimization and energy efficiency in ITS?
ii. How can a threat detection and secure routing model for ITS be designed to operate efficiently in highly dynamic environments?
iii. How can malicious traffic flow be prevented and more reliable and resilient vehicle interactions be attained without imposing communication holes?

The aim of this research is to introduce a framework for ITS using machine learning and distributed lightweight computing to detect timely threats with the integration of trust assessment and mitigating malicious devices while devices communicate. It provides energy efficiency with nominal complexity on the vehicles and an intelligent decision system for an edge-driven, resource-constrained environment. The main contributions of our research work are as follows.

i. It develops a multi-dimensional approach for trust computing for ITS with reliable and accurate decision-making strategies without incurring additional complexity on devices.
ii. Using distributed processing, a global trust model is designed and maintains the data privacy using local computing at the edges and enhances the reliability by observing trust patterns.
iii. Machine learning is integrated with a lightweight blockchain to ensure tamper-proof computing of a dynamic trust threshold based on network conditions and the level of threats. It provides security for ITS through optimized resource management by keeping potential attacks outside the communication system and enabling transparent device interaction through decentralized consensus.

The remaining sections are structured as follows. Section 2 explains the related discussion and highlights the problem gap. In Section 3, the methodology of the proposed model is explained. The experiments and their statistical analysis are described in Section 4. Finally, Section 5 concluded this work with a discussion of future work.

## 2. Related Work

The advancement of IoT systems has significantly provided the emerging solutions to support effective communication for VANETS [19,20]. They perform a key role by enabling real-time decision-making and enhancing road safety with the integration of innovative edge technologies [21,22]. It provides the enhancement for sustainable development in smart cities while maintaining urban mobility for real-time environments. Although most existing vehicular routing approaches still suffer from data reliability issues and are unable to address dynamic research challenges, they cannot provide a robust, fault-tolerant, and trust-aware model against threats [23,24]. In addition, artificial intelligence and machine learning techniques need to be explored in vehicular networks to address optimization challenges in an adaptive environment with limited constraints [25,26]. Such approaches can be useful in optimizing the system performance for the generation of latency-aware routes, enhancing the efficiency of public transportation, and fulfilling the request demands from connected devices more intelligently. They analyze the network congestion and provide more dynamic policies for traffic analysis in the public transportation system.

Authors [27] proposed a Prediction-Based Temporal Graph Routing (PT-GROUT) using a Hidden Markov Model (HMM) for Software-Defined Vehicular Network (SDVN). It provides optimal routing decisions to enhance delivery performance by utilizing temporal information from data transmissions. It effectively manages network resources by integrating dynamic programming and greedy methods to predict future network states using an HMM. Despite the dynamic nature of vehicular networks, the PT-GROUT algorithm consumes less energy and balances load distribution. The study [28] introduces a novel cluster-based dual-phase routing protocol for VANETs, integrating fog computing and Software-Defined Vehicular Networks (ICDRP-F-SDVN). The issues of traditional routing techniques have been addressed by the proposed ICDRP-F-SDVN, which enhances smooth communication and efficient resource utilization in vehicular networks. Moreover, the clustering method decreases transmission distance within intra-cluster distance devices. Only limited control overhead is imposed, thereby increasing the system’s scalability and effective routing performance.

Authors [29] propose a novel Graph-Based Trust-Enabled Routing (GBTR) for VANETs that aims to provide trustworthiness and a secure environment while devices interact and communicate. It integrates direct, indirect, and contextual trust, including factors such as location, weather, and traffic conditions. In addition, a reward is assigned to a trustworthy node, while a penalty is imposed on malicious devices; this approach supports secure routing decisions and reduces network attacks. Authors [30] address the research issue of routing disruptions and black hole attacks in VANETS using a cluster-based approach. It explores the use of artificial neural networks to identify and prevent malicious devices from participating in data transmission. Afterwards, the vehicles are clustered to improve energy efficiency and select the optimal forwarder to achieve longer connectivity routes. The traditional AODV protocol is modified to construct a routing path on demand without degrading network resources in a critical vehicular environment. Authors [31] proposed a new routing protocol for vehicular networks in sparse scenarios, inspired by the sticky bacteria algorithm. The proposed protocol makes use of various parameters, including distance, deflection angle, nearby nodes, rate difference, and road segment traffic—combining to identify the optimal routes for vehicle transmissions. Accordingly, more intelligent next-hops are selected using a sticky bacteria approach, thereby increasing the stability of connected links through consistent decision-making. The sticky bacteria approach gives the protocol a strong ability to explore and choose the best path for the vehicular network. The study [32] proposes a trust-based, energy-efficient routing method for Mobile Adhoc Networks (MANETs) by exploiting Bacteria for Aging Optimization Algorithm (BFOA), aiming to detect more optimal paths for long-run connectivity. It initiates the process by selecting cluster heads using fuzzy techniques and considers multiple factors, including direct, indirect, and recent trust values. The cluster heads are selected with high reliability to ensure a trustworthy system that enhances both network efficiency and security. Table 1 illustrates the summary and analysis of the existing studies for different metric evaluations in terms of trust model, scalability, attack handling, system response, and energy efficiency.

Accordingly, it is observed that most of the existing VANETs face communication issues in terms of link damages and inconsistent route discovery due to the rapid changes in network topology. The diverse environment not only increases the loss rate when transmitting sensitive vehicle data to processing servers, but also reduces the connectivity lifetime due to inefficient bandwidth utilization caused by high traffic. In addition, unreliable mechanisms for trust computing degrade security under critical conditions and increase the likelihood of network intrusion. Due to congestion, most approaches fail to provide timely service to interconnected devices, and malicious devices disrupt communication links by flooding them with faulty data.

## 3. Proposed Model

This section presents a detailed explanation of the proposed framework for an ITS integrated with a trust and load-balanced, energy-efficient communication system. The first component develops a trust computation scheme that leverages lightweight, multi-level device interactions to assess direct, indirect, and historical trust. Unlike most existing trusted systems that rely on cryptographic primitives and incur excessive device overhead, the proposed framework provides an intelligent, dynamic mechanism for resource-constrained edge-ITS environments and improves reliability.

### 3.1. Edge-Enabled Trust-Aware Vehicular Network

Our system model initialized the vehicles $N_{V}$, channels $N_{C}$, IoT $N_{D}$ devices, and RSUs $N_{V}$ for the formulation of the smart ITS environment along with the incorporation of trust to enhance security and network authenticity. The connected vehicles $V_{i}$ and $V_{j}$ are associated through RSU $R_{i,j}$ with communication channel $C_{i,j}$, and trust vector $T_{V}\left(t\right)$ shows the integrated trust of all the connected vehicles at time *t* using Equation (1).

(1) $$T_{V}\left(t\right)={\left[\begin{array}{cccc} T_{1}\left(t\right) & T_{2}\left(t\right) & \cdots & T_{N_{V}}\left(t\right) \end{array}\right]}^{T}$$

Similarly, the integrated trust vector $T_{integ}\left(t\right)$ of RSUs and channels is also computed to assess the individual trust for all the connected RSUs and particular channels, as defined in Equation (2).

(2) $$T_{\mathrm{combined}}\left(t\right)=\left[\begin{array}{cccccccc} T_{RSU_{1}}\left(t\right) & T_{RSU_{2}}\left(t\right) & \cdots & T_{RSU_{N_{\mathrm{RSU}}}}\left(t\right) & T_{C_{1}} & T_{C_{2}} & \cdots & T_{C_{N_{C}}} \end{array}\right]$$

In ITS, vehicles and RSUs cooperate to share road conditions, such as speed and direction, to support efficient traffic management. In cases of high interference and congestion, it can lead to delays in receiving information or even result in a loss of accuracy. Thus, the proposed framework defines the optimization problem by minimizing total interference between vehicles and RSU pairs using Equation (3).

(3) $$\begin{array}{cc} \underset{C_{k}}{\mathrm{minimize}}\; & \sum_{i=1}^{N_{V}}\sum_{j=1}^{N_{RSU}}{\mathrm{Interference}}_{ij}\\ \mathrm{subject}\;\mathrm{to}\; & \gamma_{ij}\left(t\right)\geq \theta ,\;\forall \;i,\;j,\;t \end{array}$$

The proposed framework determines the aggregated trust score of vehicle *i* at time *t* by integrating direct $T_{\mathrm{direct}}\left(t\right)$, indirect $T_{\mathrm{indirect}}\left(t\right)$, and historical trust $T_{\mathrm{historical}}\left(t\right)$ based on Equation (4).

(4) $$T_{i}\left(t\right)=\left[\begin{array}{ccc} \alpha & \beta & \gamma \end{array}\right]\left[\begin{array}{c} T_{\mathrm{direct}}\left(t\right)\\ T_{\mathrm{indirect}}\left(t\right)\\ T_{\mathrm{historical}}\left(t\right) \end{array}\right]$$

where α,β,γ≥0 are non-negative weights with uniform contribution in the evaluation of trust score, i.e., α+β+γ=1. The direct distance is determined from vehicle *i* to either vehicle *j* or $RSU_{j}$ based on the ratio of past successful interactions $N_{\mathrm{success}}\left(t\right)$ at time *t* as defined in Equation (5).

(5) $$T_{\mathrm{direct}}\left(t\right)=\frac{N_{\mathrm{success}}\left(t\right)}{N_{\mathrm{total}}\left(t\right)}$$

where $N_{\mathrm{total}}\left(t\right)$ denotes the total number of interactions. On the other hand, the indirect trust $T_{\mathrm{indirect}}$ presents a trust score for device (vehicle/RSU) *j* by exploring the recommendations provided by third trusted parties *k*, as given in Equation (6).

(6) $$T_{\mathrm{indirect}}\left(t\right)=\frac{\sum_{k=1}^{m}T_{ik}\left(t\right)\cdot T_{kj}\left(t\right)}{\sum_{k=1}^{m}T_{ik}\left(t\right)}$$

Moreover, to preserve historical trust, the proposed model employs an exponential moving average. It enhances the network security against threats with intelligent monitoring of device behavior, as defined in Equation (7). Accordingly, IoT systems deliver consistent, trusted decisions for network collaboration and task offloading to deliver network services.

(7) $$T_{\mathrm{historical}}\left(t\right)=\lambda \;T_{\mathrm{historical}}\left(t-1\right)+\left(1-\lambda \right)\;T_{\mathrm{current}}\left(t\right)$$

where $T_{\mathrm{historical}}\left(t-1\right)$ denotes previous historical trust, the latest trust observation at time *t* is $T_{\mathrm{current}}\left(t\right)$, and λ (0<λ<1) is the decay to manage the influence of device history with recent observations. When λ is high, trustworthiness communication is primarily based on the device’s past behavior; otherwise, it updates rapidly based on recent device activity. The local score is evaluated for each vehicle *i* using Equation (8), combining the direct, indirect, and historical trust components with locally observed interaction data; thus, the proposed framework preserves data privacy at network edges.

(8) $$T_{i}^{\mathrm{local}}\left(t\right)=\frac{\alpha T_{direct}^{i}\left(t\right)+\beta T_{indirect}^{i}\left(t\right)+\gamma T_{historical}^{i}\left(t\right)}{\alpha +\beta +\gamma }$$

RSUs are performing the role of local edges and intermediate aggregators to collect the local trust scores $T_{i}^{local}\left(t\right)$ of all the vehicles that fall in their coverage area and formulate a global trust model $T^{\mathrm{global}}\left(t\right)$ using edge-level distributed capabilities and enhancing trust-awareness in the system as defined in Equations (9) and (10).

(9) $$T_{e}\left(t\right)=\frac{1}{|V_{e}|}\sum_{i\in V_{e}}T_{i}^{local}\left(t\right)$$

(10) $$T^{\mathrm{global}}\left(t\right)=\frac{1}{\left|E\right|}\sum_{e\in E}T_{e}\left(t\right)$$

### 3.2. Blockchain-Integrated Global Trust Model

In the next phase, the proposed framework maintains a global trust record in a lightweight blockchain. It provides system transparency along with integrity of devices and data privacy, as given in Equation (11).

(11) $$B\left(t\right)=\left\{T^{\mathrm{global}}\left(t\right),\;\mathrm{Hash}\;\left(T^{\mathrm{global}}\left(t-1\right)\right),\;t\right\}$$

where *t* is the timestamp of the trust update and reserves the chronological order of blockchain entries, while Hash(·) denotes a hashing function to ensure the immutability of previous global trust values. Moreover, computed hashes are interlinked for consecutive blocks to enhance consistency and trust in the vehicular network, as given in Equation (12).

(12) $$\mathrm{Verify}\left(B(t)\right)=\left\{\begin{array}{cc} 1, & \mathrm{if}\;\mathrm{Hash}(B(t-1))=B(t).\mathrm{prevHash}\\ 0, & \mathrm{otherwise} \end{array}\right.$$

**Figure 1:**
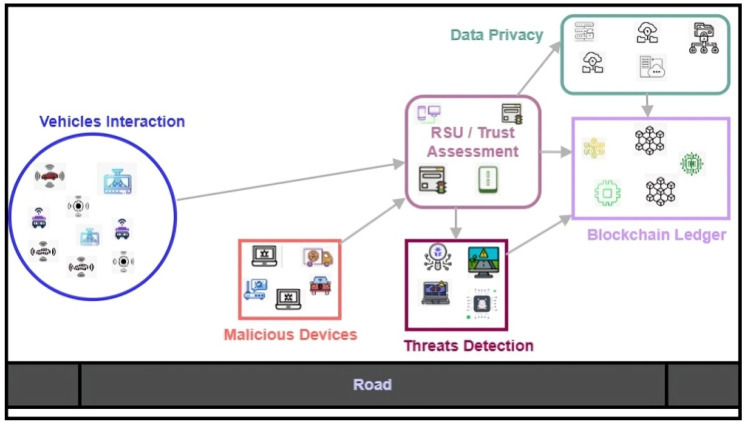
System architecture of vehicular trust management integrated blockchain security.

Figure 1 illustrates the system architecture of trustworthy and secured vehicular networks that comprise edges/RSU with intelligent capabilities to identify malicious threats in a real-time environment. The security integrated with the blockchain ledger verifies the device’s authenticity and provides transparent communication, along with data integrity. The combination of learning modules and determining untrustworthy devices in the proposed model enhances data protection and maintains the resilience of the system against network threats with energy efficiency.

**Figure 2:**
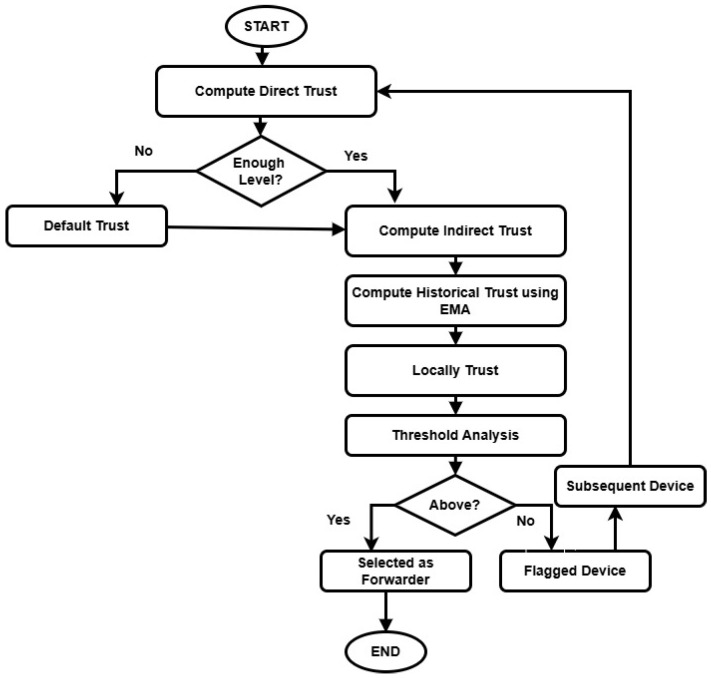
Working Flow of Trust Evaluation Process in Distributed Vehicular Systems.

Figure 2 depicts the procedure of trust computation using multi-level parameters. It comprises direct, indirect, and historical trust and enhances reliable communication for vehicular networks. Using the exponential moving average technique, the historical trust is maintained to reduce the probability of risks while identifying the forwarder node in traffic management. Moreover, the devices with low trust scores are flagged and generate an event to route updates and reformulation.

**Figure 3:**
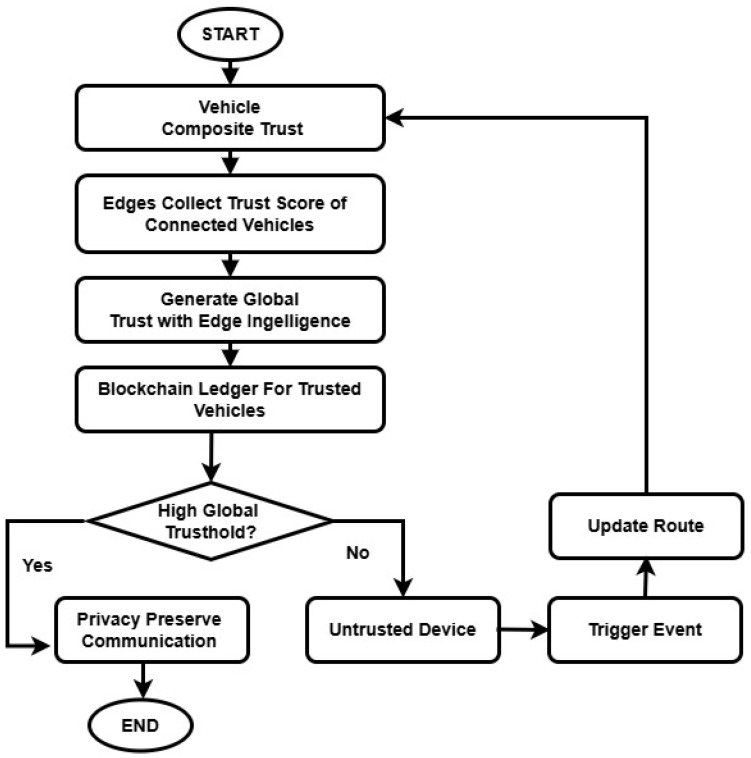
Working Flow of Security Measurement with Trust Assessment Process.

Figure 3 illustrates the intelligence of edges to determine the global trust model. All the vehicles compute their trust scores and send the local information towards the nearest RSU for performance aggregation and generate a global trust communication. The RSU maintains a blockchain ledger of the trust score, thus providing more secure and tamper-resistant communication while verifying the vehicle’s authenticity in IoT-based collaborative environments.

**Figure 4:**
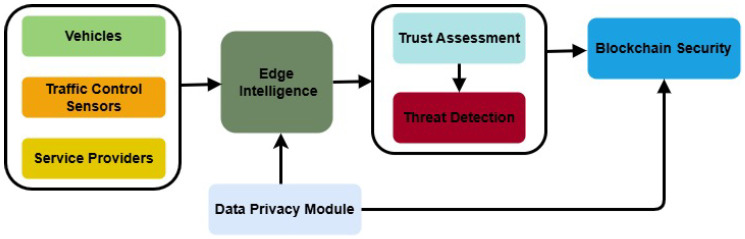
Components in Proposed Model for AI-enabled Trust Evaluation in Vehicular Systems.

Figure 4 depicts the flow of the developed components in the proposed model. The vehicles, wireless sensors, and various service providers are interconnected to each other for collecting the road and traffic information. After data aggregation, it is transmitted to the edges/RSU for local processing and to make a decision for trust assessment and threat detection. The trust is dynamically updated by utilizing the device’s behavior with respect to time and maintaining a global trust model at the edges. In addition, a blockchain ledger is employed to securely send the device information and store the computed trust scores in a transparent manner, along with maintaining the data privacy of the vehicular network.

Algorithm 1 illustrates the mechanism of interconnection of vehicles with smart devices and sensors to compute the local trust for ITS. The computed trusts are processed at the network edge, lowering the overhead on the devices for attaining optimized connectivity. The trust is determined based on direct, indirect, and historical methods, and edges explore their coverage region to aggregate trust scores from vehicles in a weighted manner, along with the uniform contribution of the coefficients. Due to the involvement of edges, it reduces the computational load on the ITS and enhances the reliability for sensitive and private communication. Algorithm 2 depicts the working procedure of the proposed model to determine the global trust model by aggregating the local trust of each vehicle by the edges. The long-term trust patterns are obtained using global trust and stored in the form of a blockchain ledger along with the interconnection of blocks using hashes. It provides transparent and tamper-resistant communication for devices and enhances a reliable decision-making system with data integrity and network authenticity.
**Algorithm 1**
Local Trust Computation Using Optimized Weighted Scheme**Require:** Direct interactions *D*, recommendations *R*, previous historical trust $T_{hist}\left(t-1\right)$ **Ensure:** Local trust score $T_{i}^{\mathrm{local}}\left(t\right)$  1:Compute direct trust: $T_{direct}\leftarrow \frac{N_{success}}{N_{total}}$  2:Compute indirect trust using weighted recommendations from *R*  3:Update historical trust: $T_{hist}\left(t\right)\leftarrow \lambda T_{hist}\left(t-1\right)+\left(1-\lambda \right)T_{current}$  4:Compute local trust:$$T_{i}^{\mathrm{local}}\left(t\right)\leftarrow \alpha T_{direct}+\beta T_{indirect}+\gamma T_{hist}\left(t\right)$$  5:$T_{i}^{\mathrm{local}}\left(t\right)$ to [0,1]  6:**return** $T_{i}^{\mathrm{local}}\left(t\right)$

**Algorithm 2** Edge-Assisted Global Trust Aggregation with Blockchain Assurance**Require:** Local trust values $\left\{T_{i}^{\mathrm{local}}\left(t\right)\right\}$ from vehicles, edge set E
**Ensure:** Secure global trust value $T^{\mathrm{secure}}\left(t\right)$
  1:**for** each edge node e∈E **do**  2:    Initialize sum ←0  3:    **for** each vehicle $i\in V_{e}$ **do**  4:     sum ← sum $+\;T_{i}^{\mathrm{local}}\left(t\right)$  5:    **end for**$$T_{e}\left(t\right)\leftarrow \frac{1}{|V_{e}|}\sum_{i\in V_{e}}T_{i}^{\mathrm{local}}\left(t\right)$$  6:**end for**$$T^{\mathrm{global}}\left(t\right)\leftarrow \frac{1}{\left|E\right|}\sum_{e\in E}T_{e}\left(t\right)$$  7:Create blockchain block $B\left(t\right)\leftarrow \{T^{\mathrm{global}}\left(t\right),\mathrm{Hash}\left(B\left(t-1\right)\right),t\}$  8:Append B(t) to the blockchain ledger  9:Verify block integrity using hash linkage10:Set secure trust value:$$T^{\mathrm{secure}}\left(t\right)\leftarrow T^{\mathrm{global}}\left(t\right)$$11:**return** $T^{\mathrm{secure}}\left(t\right)$


## 4. Result Discussion

This section presents and evaluates the assessment of the proposed model against GBTR and BFOA using the NS-3 network simulator. The experiments are analyzed over varying node speeds and pause times. The simulated environment is equipped with 200–1000 vehicle sensors for continuous sensing of road and traffic conditions along with RSUs and edges. The edges act as local decision-makers, performing local processing to reduce the additional load on the device and improve response times for vehicles. The TensorFlow and PyTorch libraries were used for AI modeling and training; the modeling implementation provided secure transmission and reliable protocols through the NS-3 Security Module. The simulation parameters are defined in Table 2. In this research work, we generated and explored the synthetic data to replicate the performance of the proposed model in realistic transportation and security scenarios. We run the simulation many times, and the recorded dataset is comprised of 60,000 records gathered from various IoT across 200–1000 vehicles. The individual sample consists of device identity, its behavior, trust metric, threat indicators, and resource consumption. Table 3 defines the schema of our synthetic dataset to evaluate the training and learning process of the proposed model.

**Figure 5:**
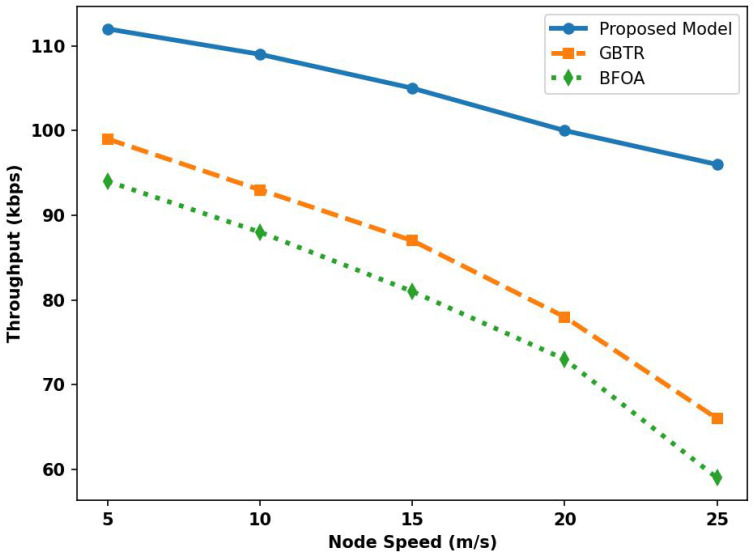
Analysis of throughput for proposed model, GBTR, and BFOA over node speed (5–25 m/s).

Figure 5 illustrates the performance of the proposed model, GBTR, and BFOA for network throughput over varying node speeds. Based on the results, the proposed model improved throughput by 50% and 62% compared with existing studies. It integrates intelligent routing methods with adaptive selection of forwarders near damaged links and compromised devices. Ultimately, it enhances the delivery ratio of the network and minimizes the retransmission in varying conditions. The reduction in retransmissions not only improved throughput in the congested ITS network but also made the system more available for real-time monitoring and communication with end users about road conditions and traffic management. Moreover, malicious devices are detected in real time using lightweight security strategies, ensuring reliable links for transmitting massive vehicle data to the intended destination.

**Figure 6:**
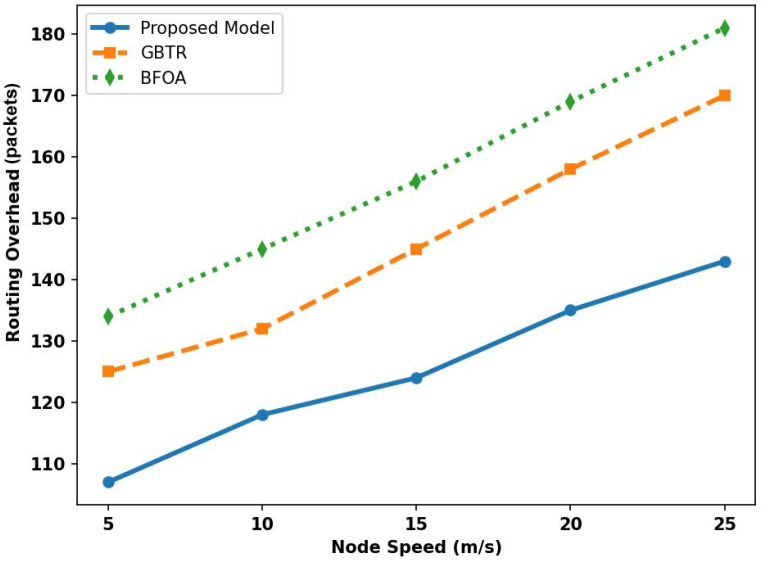
Analysis of overhead for proposed model, GBTR, and BFOA over node speed (5–25 m/s).

In Figure 6, the performance of the proposed model and existing approaches is compared in terms of overheads over varying node speeds. Due to the intelligent monitoring and data gathering techniques, the proposed model reduces the transmission of duplicate data packets among the vehicles and lowers the overhead on the devices, thus improving overhead by 34% and 39% compared to related approaches. Moreover, the routes are more stable and reduce the time to connect traffic in the ITS, so their reformulation is not frequently generated. Using AI modeling, the load is nearly optimally distributed across routes to strengthen the communication system, limit the propagation of control messages, and thus reduce the risk of control-message flooding and link overload.

**Figure 7:**
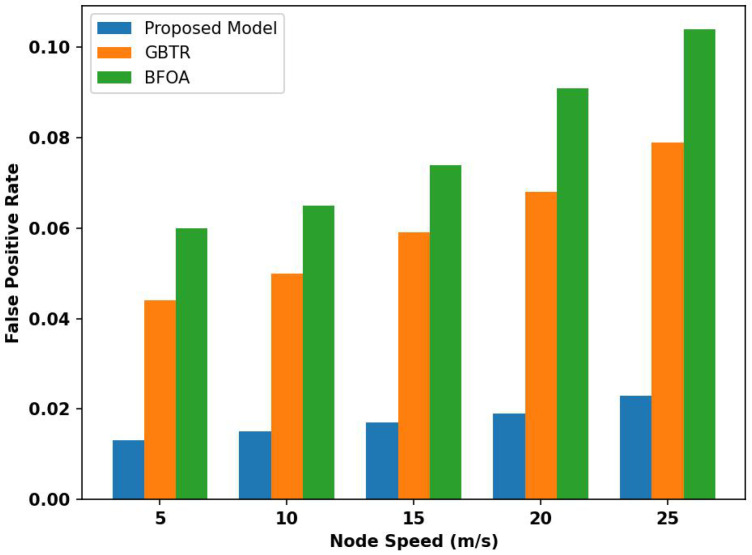
Analysis of false positive rate for proposed model, GBTR, and BFOA over node speed (5–25 m/s).

Figure 7 compares the proposed model with existing approaches in terms of the false positive rate across varying node speeds. Based on the statistical analysis, it was observed that the proposed model reduces the false positive rate by 67% and 69% compared to GBTR and BFOA. It is due to the combination of trust measurement, which reduces the likelihood of malicious activity and data compromise. The intelligent module of the proposed model minimizes the network threats to affect the data integrity and prevents the fault traffic from flooding the erroneous transmission on the communication links. Moreover, by regularly monitoring network conditions and device activities, the proposed model enables early detection of network threats through positive learning and effectively reduces misclassification between normal and attacker behaviors.

**Figure 8:**
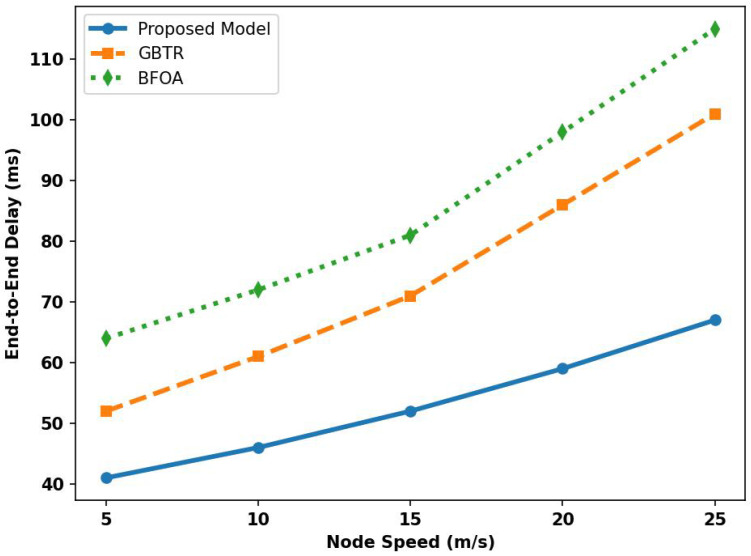
Analysis of delay rate for proposed model, GBTR and BFOA over node speed (5–25 m/s).

Figure 8 depicts the latency performance of the proposed model with GBTR and BFOA across varying node speeds. According to the obtained results, the proposed model improved the latency rate of the proposed model over existing studies by 33% and 38%. It is due to detecting routing holes and preventing malicious threats from transmitting bogus packets on the links. Reducing retransmissions between connected vehicles reduces congestion and unnecessary traffic. The proposed model effectively delivers the required data to the destination in a timely manner. Reliable data forwarders enable the generation of an alternative route that is much more efficient and ultimately route vehicles’ data, along with road conditions, in the minimum time frame. In addition, edge-based task offloading also offers local processing and avoids processing the ITS request on a centralized cloud-based platform.

**Figure 9:**
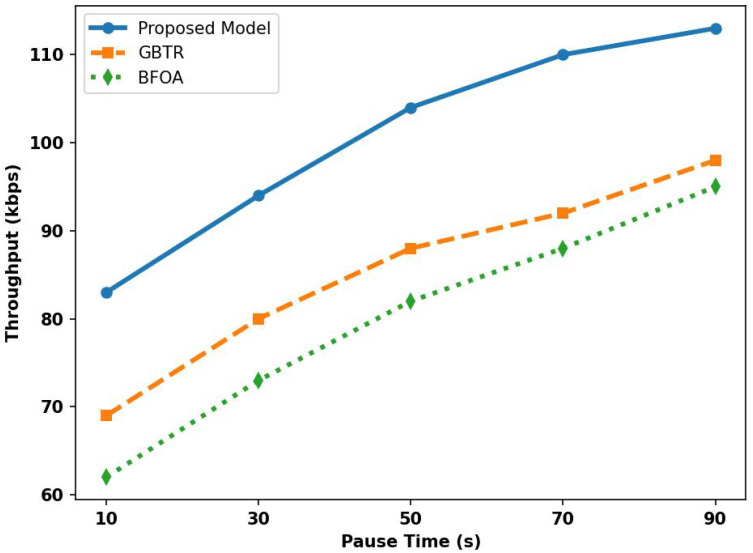
Analysis of throughput for proposed model, GBTR and BFOA over pause time (10–90 ms).

In Figure 9, the proposed model is analyzed with GBTR and BFOA for network throughput in terms of varying pause time. The results showed that the proposed model achieved improvements of 43% and 50% over related schemes. It is due to the adaptive selection of data forwarders by exploring network conditions and device traffic patterns and to ensuring consistent throughput while reducing route damage when vehicles pause. The proposed model reschedules data transmission using effective strategies whenever a vehicle pauses for longer or shorter durations, thereby achieving higher network throughput than the GBTR and BFOA schemes. Moreover, the trust is updated dynamically and provides more reliable routes for the ITS environment to support device interaction and real-time data sharing, thereby enabling rapid route reformulation in the event of any malicious behavior.

**Figure 10:**
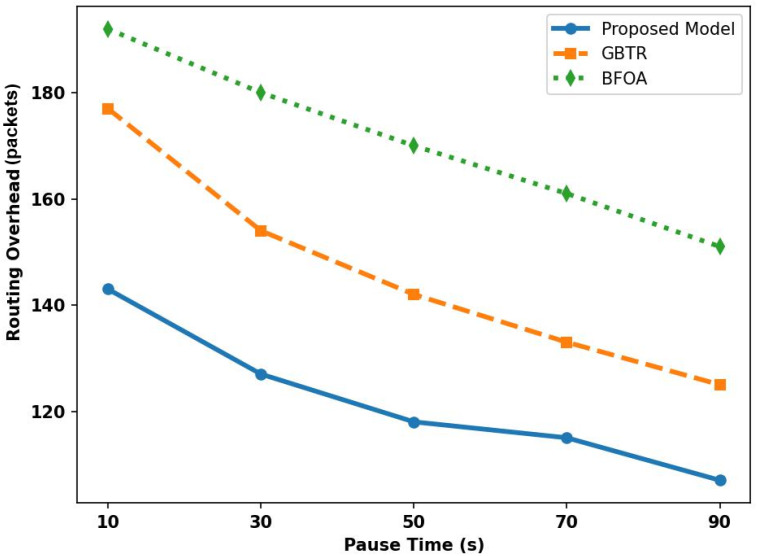
Analysis of overhead for proposed model, GBTR, and BFOA over pause time (10–90 ms).

Figure 10 depicts the performance evaluation of the proposed model and benchmark approaches for overhead under varying pause times. By exploring multiple parameters and continuously observing device behavior, the proposed model makes the constructed routes more consistent and balances traffic load across routes. It reselects forwarders based on the devices’ learned patterns, reducing overhead by 31–35% compared with existing approaches. In addition, reducing retransmissions limits the flood of control messages and imposes the least overhead on the ITS environment. Unlike existing models, which are constrained by pause times among vehicles, the proposed model adjusts the transmission of control messages and reduces the frequency of topology changes, thereby controlling device load and preventing route failures.

**Figure 11:**
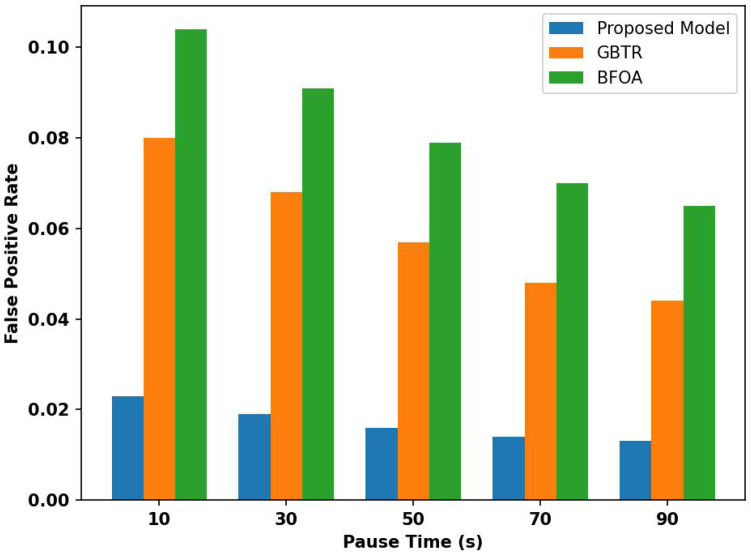
Analysis of false positive rate for proposed model, GBTR, and BFOA over pause time (10–90 ms).

Figure 11 evaluates the performance of the proposed model with existing techniques for the false positive rate under varying pause times. Based on the results, it was noticed that the proposed model improves it by 71% and 69% as compared to existing schemes due to the anomaly detection method and reduces the disruption among devices for effective data delivery. The trust computation and its dynamic adjustment prevent the misclassification of network threats and guarantee secure communication between devices. It decreases the ratio of false decision-making due to the intelligent behavior of the proposed model by exploring an AI-driven approach. It continuously exploits feedback criteria to attain a learning process. It makes the ITS persistent in detecting threats on time while decreasing the probability of detecting network behavior as false positives.

**Figure 12:**
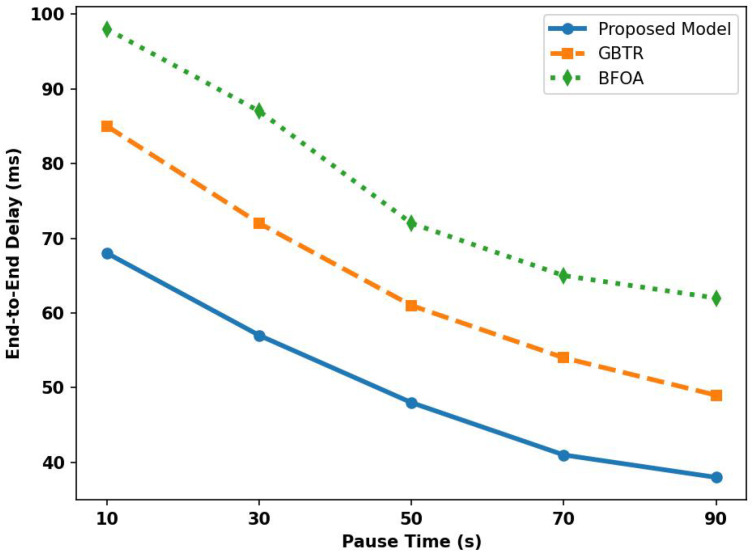
Analysis of delay rate for proposed model, GBTR, and BFOA over pause time (10–90 ms).

In Figure 12, the proposed model is assessed for latency in the comparison of existing schemes under varying pause times. Based on the statistical analysis, the proposed model improves the latency performance by 42% and 48%, respectively, due to the integration of edge-level computing and performing task offloading on the nearest edges. Unlike GBTR and BFOA, over a longer pause time, the proposed model maintains the stable network topology based on the AI-driven learning capabilities while generating routing paths, thereby resulting in improved end-to-end connections and increasing the stability period of the established links. In addition, the detection of unauthentic behavior of the devices on time leads to the reformulation of routing paths. It prevents bottlenecks of communication holes, ultimately improving the latency risks between associate vehicles. The local processing at the edge level in the proposed model also improved the integrity of the incoming request, and only authentic devices are allowed to be a part of the blockchain ledger for interaction with the cloud platform and to optimize the resources without additional delay in network mobility.

Table 4 depicts the statistical analysis of the performance results for the proposed model, GBTR, and BFOA against varying node speed and pause time for several simulations. To evaluate the performance and statistical validation of the proposed model, we run 40 simulations. Based on the results, the proposed model shows consistent and substantial improvements over baseline approaches across multiple metrics. Moreover, we compute the reproducibility score to indicate that results are not random and reliable by running the simulation multiple times; over 95% of samples generate outcomes within ±5% of the mean.

## 5. Conclusions

In a modern system, ITS is integrated with the IoT system, performing a vital role while monitoring and maintaining road safety. The vehicle and smart infrastructure share the real-time data and traffic conditions with the RSU for further analysis and transmit it toward edge-level processing. The dynamic infrastructure of ITS imposes various research challenges in data sensing, resource efficiency, and network convergence. Moreover, trust is a significant research issue in data security against threats. We introduced an edge-driven, trust-aware model for vehicular networks to provide a trustworthy communication environment and optimal routing. It develops a fault-tolerant and robust technique to authenticate the devices and enhances the reliability against potential network attacks. The trust is updated in a timely manner based on network conditions and vehicle behavior to mitigate threats to data privacy and integrity. In addition, blockchain is integrated to provide more transparent and tamper-proof communication in unpredictable circumstances. The simulation-based results reveal remarkable performance of the proposed model compared to GBTR and BFOA in terms of throughput, delay, overhead, and false positive rate. However, the proposed model imposes additional energy constraints and computational cost for trust management when increasing to large-scale vehicular networks. The overall system efficacy may degrade when balancing resource utilization across dedicated network tasks. For future research, we plan to enhance our proposed model with a deep learning component to predict distributed threats while imposing minimal overhead on constrained devices. Furthermore, its effectiveness needs to be tested at scale across a large network region to ensure scalability and preserve privacy.

## Figures and Tables

**Table 1:** Critical Comparison of Trust-based Secured Routing Schemes in VANETs.

Existing Study	Proposed Methods	Trust Model	Attack Handling	Scalability	Mobility Support	Response Time	Energy Efficiency	Demerits
PT-GROUT [27]	HMM-based prediction on temporal graph	No trust	Path reliability	Moderate	High	High	Moderate	Depends heavily on prediction accuracy; computational cost
ICDRP-F-SDVN [28]	Cluster-based dual-phase with Fog/SDN	No explicit trust	Improved robustness, no explicit trust focus	Good	Good	Moderate	Improved	Complex cluster management; possible delays
GBTR [29]	Combined multiple trust methods	Multi-source trust	Identifies and avoids malicious nodes	Moderate	High	High	Moderate	Overhead due to frequent trust computation; may occur sub-optimal decisions
Artificial Neural Network (ANN)-based Cluster Routing [30]	ANN-based malicious detection	Direct/Indirect trust	Detects malicious/black hole nodes with ANN	Good	Good	High	Moderate	Needs training data; ANN computation overhead
Sticky Bacteria Routing [31]	Metaheuristic	No explicit trust	Favors stable links, not attacks	Moderate	High	Moderate	Moderate	Parameter and algorithm tuning; limited dynamic adaptation
BFOA Trust Routing [32]	Bacterial foraging and fuzzy clustering	Multi-parameters trust	Trust-based secure multi-hop routing	Moderate	Good	Moderate	Improved	Complex optimization; trust model overhead

**Table 2:** Simulation Parameters.

Parameter	Value
Simulation tool	NS-3
Vehicle sensors	200–1000
Simulation runs	40
1 RSU	100–200 vehicles
Transmission range	3 m
libraries	TensorFlow and PyTorch
CPU configuration	Intel Core i7
Initial energy	5J
Malicious nodes	10–30
Node speed	5–25 m/s
Pause time	10–90 ms
Weights selection	$\alpha =\beta =\gamma =\frac{1}{3},\;\mathrm{where}\;\alpha +\beta +\gamma =1$
Performance scenarios	Node speed and Pause time
Evaluation metrics	Network throughput, latency, routing overhead, false positive rate

**Table 3:** Schema of Synthetic Dataset for the Assessment of Proposed Model.

Feature	Description	Unit
device_id	Unique identifier	–
reading_timestamp	Date and time when the sensor reading was recorded	–
data_value	Measured sensor value	Varies
cpu_usage	CPU usage percentage of the edge/IoT device	%
memory_usage	Memory consumption percentage	%
energy_consumption	Energy depletion	J
trust_score	Computed trust score using reliability and network behavior	[0–1]
latency	Communication delay	ms
packet_loss_rate	Packet loss ratio	%
anomaly_score	Score indicating potential threat	[0–1]
threat_label	Truth values (0: Normal, 1: Attack)	Binary

**Table 4:** Performance analysis of the Proposed model and benchmark schemes with the assessment of reproducibility.

Scenario	Metric	Over GBTR (%)	Over BFOA (%)	Reprod. Score (%)
**Node Speed**	Throughput	50.0	62.5	97.2
	End-to-End Delay	33.3	37.5	96.4
	Routing Overhead	34.0	38.7	96.8
	False Positive Rate	67.9	68.5	95.5
**Pause Time **	Throughput	42.9	50.0	97.0
	End-to-End Delay	41.8	48.4	96.6
	Routing Overhead	30.5	35.3	96.0
	False Positive Rate	71.0	70.0	94.8

## Data Availability

All data is available in the manuscript.

## Abbreviations

The following abbreviations are used in this manuscript:

IoT	Internet of Things
ITS	Intelligent Transportation System
VANETs	Vehicular Adhoc Networks
RSU	Roadside Unit
GBTR	Graph-Based Trust-Enabled Routing
BFOA	Bacteria for Aging Optimization Algorithm
PT-GROUT	Prediction-Based Temporal Graph Routing
MANETs	Mobile Adhoc Networks
ANN	Artificial Neural Network
